# Fluconazole‐Induced Fixed Drug Eruption With Cross‐Reactivity to Clotrimazole—Confirmation With Patch Testing

**DOI:** 10.1155/crdm/1152541

**Published:** 2026-01-28

**Authors:** D. S. Sron, P. Martinez, J. Iacobelli, P. Singh, B. M. Ricciardo

**Affiliations:** ^1^ Department of Dermatology, Fiona Stanley Hospital, Murdoch, Western Australia, Australia, fsh.health.wa.gov.au; ^2^ Department of Immunology, Fiona Stanley Hospital, Murdoch, Western Australia, Australia, fsh.health.wa.gov.au; ^3^ Pathwest QEII, Nedlands, Western Australia, Australia; ^4^ University of Western Australia, Crawley, Western Australia, Australia, uwa.edu.au

**Keywords:** case report, cross-reactivity, fixed drug eruption, fluconazole

## Abstract

First described in 1994, fixed drug eruption (FDE) to fluconazole is uncommon but possibly underdiagnosed. Of these, women with vaginal candidiasis remain the most affected, with on average more than four occurrences prior to diagnosis. We present a case of a 29‐year‐old female who presented after her third episode of an itchy, oedematous, blistering rash on her right hand that developed 2 h following ingestion of 150 mg of fluconazole. She reported two similar episodes in the 2 years prior, all following administration of fluconazole for vaginal candidiasis. Each episode resulted in a rash localized to her right hand, with each subsequent exposure resulting in faster onset of symptoms and signs. A FDE to fluconazole was suspected clinically, and lesional skin biopsies were consistent with this. The diagnosis was confirmed with a positive patch test to 5% fluconazole applied to the affected skin on the right hand. Cross‐reactivity with clotrimazole was confirmed with a positive patch test to clotrimazole 5%. She was subsequently advised to avoid both fluconazole and clotrimazole. Although cross‐reactivity between different azole antifungal agents has been described, cross‐reactivity between fluconazole and clotrimazole is a novel finding. This case raises awareness of FDE to fluconazole, in particular for women being treated for vaginal candidiasis, and highlights the importance of patch testing to other antifungal agents to assess for cross‐reactivity.

## 1. Case Report

A 29‐year‐old female presented following her third episode of a pruritic rash localized to her right hand, developing two hours following ingestion of 150 mg of fluconazole. She reported two similar episodes in the 2 years prior, all following administration of oral fluconazole for vulvovaginal candidiasis. Each episode resulted in an annular dermal plaque localized to the first interdigital webspace of her right hand (Figure 1), with subsequent exposures resulting in faster onset. Her background medical history included Crohn’s disease (treated with vedolizumab) and atopic dermatitis (well controlled with topical therapy). Fixed drug eruption (FDE) to fluconazole was suspected clinically, and lesional skin biopsies taken for histopathology revealed mixed inflammatory changes that were supportive of a drug/medication reaction (Figure 2). Fluconazole was ceased, and she was treated with betamethasone dipropionate 0.05% ointment twice daily until clear.

**Figure FIGURE 1:**
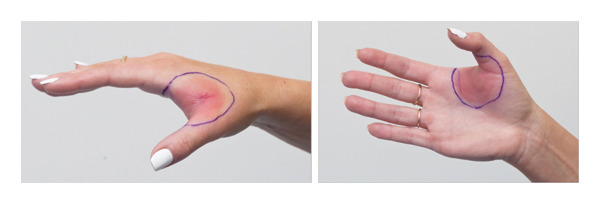
Annular dermal plaque localized to the first interdigital webspace of the patient’s right hand.

**Figure FIGURE 2:**
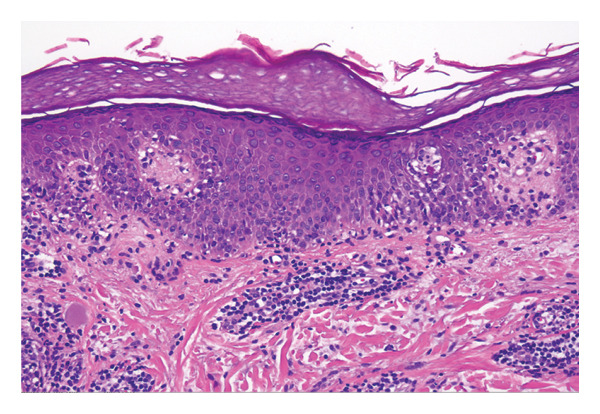
The biopsy reveals acral‐type keratin overlying mildly acanthotic epidermis in which there is variable spongiosis and interface inflammatory change. There is an underlying superficial and mid‐dermal perivascular inflammatory infiltrate comprising lymphocytes, histiocytes, and occasional eosinophils. Significant numbers of dermal melanophages are not seen. Overall, the findings support a drug reaction.

FDE was confirmed with patch testing to 5% fluconazole petrolatum (pet.), 5% clotrimazole pet., and a pet. control applied on separate 8 mm Finn chambers to lesional skin 3 months after presentation (Figure 3(a)). Clotrimazole was chosen as a potential topical alternative for treatment. Readings on Day 2 showed positive reactions to 5% fluconazole pet. and 5% clotrimazole pet (Figure 3(b)). A control series of identical patches applied to the patient’s back was negative. Avoidance of both fluconazole and clotrimazole was advised, along with follow‐up with her treating physician to advise on alternative treatments for vulvovaginal candidiasis to guide subsequent sensitivity testing.

Figure FIGURE 3(a) Patch tests application of 1—pet. Control, 2%–5% fluconazole pet. 3%—5% clotrimazole pet. (b) Positive patch test reading on day two to fluconazole and clotrimazole indicated by dermal erythematous plaques. Note the presence of two scars from biopsy sites taken near site 1.

**Figure:**
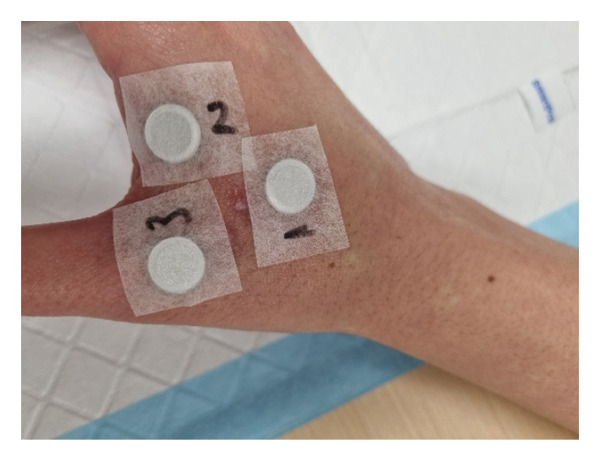
(a)

**Figure:**
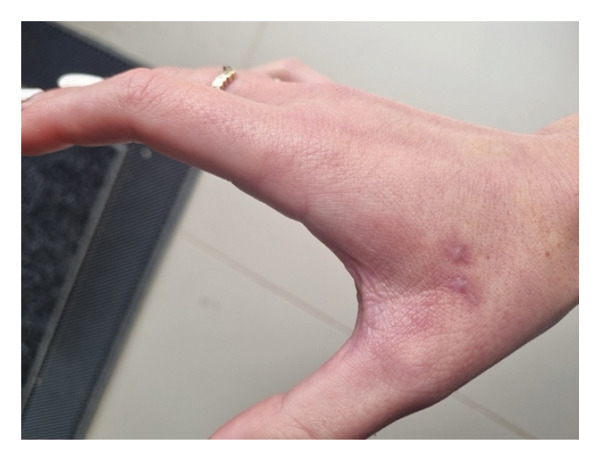
(b)

## 2. Discussion

FDE secondary to fluconazole is uncommon, with only 30 cases published in the medical literature. Females accounted for more than 75% of reported cases, predominantly occurring in the context of fluconazole prescribed for the treatment of vulvovaginal candidiasis [1]. For affected males, the indication for fluconazole treatment included candida balanitis, oral candidiasis, tinea corporis, and tinea cruris. Fluconazole‐induced FDE may affect the hands, face, trunk, and limbs, noting there are two reported cases affecting the interdigital webspace as in our case [2, 3]. A bullous variant of FDE to fluconazole has been described in nine cases [4].

FDE represents a Type IV hypersensitivity reaction resulting in the reactivation of the CD8+ memory T cells residing in the basal layer of the epidermis [5]. Classic annular erythematous plaques, often with vesiculobullous formation, that heal with postinflammatory hyperpigmentation are seen [6]. Memory T‐cells remain dormant, and re‐exposure leads to a repeat immune response, typically with more sudden and severe clinical features. The presence of memory T cells causes positive patch testing on lesional skin, with negative findings on unaffected skin [6].

FDE is typically a clinical diagnosis based on a consistent temporal history of drug exposure and examination findings. Histopathology can aid in confirming the diagnosis, along with patch testing, oral provocation testing, and, in rare cases, lymphocyte transformation testing [7].

Due to the structural similarities between different azole antifungals, cross‐reactivity remains a possibility. Variability in cross‐reactivity is likely due to differences in molecular structure and side chains [8]. Cross‐reactivity with fluconazole (a triazole) has been reported with itraconazole (a nitroimidazole) and ornidazole and tinidazole (both nitroimidazoles) [7, 9, 10]. One report also suggested cross‐reactivity between fluconazole and clotrimazole (a phenethyl imidazole), in which a patient with a patch test confirmed allergic contact dermatitis to clotrimazole subsequently developed a widespread exanthema 48 h after fluconazole exposure [11]. The authors postulated systemic allergic dermatitis to fluconazole, though patch testing was not performed to confirm this.

The findings of the current case are extremely rare, particularly given the structural dissimilarity between the agents, a triazole (fluconazole) and clotrimazole (an imidazole). Cross‐reactivity may reflect immune recognition of shared antigenic determinants, where the reaction is not driven by the azole ring itself but by common reactive metabolites that form hapten‐protein complexes. These complexes are immunogenic and trigger CD8+ T cells that are mediated in the Type IV hypersensitivity response [12].

Further research is needed to determine the exact mechanisms of cross‐reactivity but due to the unpredictable nature, caution should be taken with systemic or topical azole re‐exposure after an azole‐induced FDE [12].

First‐line treatments for acute vulvovaginal candidiasis include intravaginal clotrimazole cream (1% applied via applicator for 6 nights, 2% via applicator for 3 nights, or 10% via applicator for 1 night) or pessary (100 mg intravaginally for 6 nights or 500 mg once only) and oral fluconazole (150 mg as a single dose or as two doses on Days 1 and 4 for severe infection) [13]. Where these medications are contraindicated, alternatives include intravaginal nystatin cream (100,000 units/5 g applied via applicator for 14 nights) and oral intraconazole (100 mg twice daily for 1 day or 100 mg once daily for 3 days) [14].

We present an uncommon case of FDE to fluconazole in a female patient treated for vulvovaginal candidiasis to raise clinician awareness. We report the first case of patch‐test confirmed cross‐reactivity with clotrimazole in the setting of fluconazole‐induced FDE.

## Funding

No funding was received for this manuscript.

## Consent

Written patient consent was obtained for the use of the patient’s clinical history and images.

## Conflicts of Interest

The authors declare no conflicts of interest.

## Data Availability

No data were needed for sharing in this article.
